# The Expanding Role for Retinoid Signaling in Heart Development

**DOI:** 10.1100/tsw.2008.39

**Published:** 2008-02-25

**Authors:** Loretta L. Hoover, Elizabeth G. Burton, Bonnie A. Brooks, Steven W. Kubalak

**Affiliations:** Department of Cell Biology and Anatomy, Cardiovascular Developmental Biology Center, Medical University of South Carolina, Charleston, SC, USA

**Keywords:** retinoic acid receptor (RAR), retinoid X receptor (RXR), retinoids, signaling, embryo, heart, cardiac development

## Abstract

The importance of retinoid signaling during cardiac development has long been appreciated, but recently has become a rapidly expanding field of research. Experiments performed over 50 years ago showed that too much or too little maternal intake of vitamin A proved detrimental for embryos, resulting in a cadre of predictable cardiac developmental defects. Germline and conditional knockout mice have revealed which molecular players in the vitamin A signaling cascade are potentially responsible for regulating specific developmental events, and many of these molecules have been temporally and spatially characterized. It is evident that intact and controlled retinoid signaling is necessary for each stage of cardiac development to proceed normally, including cardiac lineage determination, heart tube formation, looping, epicardium formation, ventricular maturation, chamber and outflow tract septation, and coronary arteriogenesis. This review summarizes many of the significant milestones in this field and particular attention is given to recently uncovered cross-talk between retinoid signaling and other developmentally significant pathways. It is our hope that this review of the role of retinoid signaling during formation, remodeling, and maturation of the developing heart will serve as a tool for future discoveries.

## INTRODUCTION

The early work of Warkany and colleagues, demonstrating the effects of vitamin A deficiency on embryos of pregnant rats, laid the groundwork for investigating the significance of retinoic acid (RA) signaling during heart development[1,2,3]. Among the experimental models employed to study the role of retinoids in development are avian and mammalian systems. Using these models, experimental strategies have utilized both retinoid deficiency and excess, and have shown that a variety of developmental processes during cardiac development are either dependent on or are influenced by retinoid signaling. Even though the realization that vitamin A levels profoundly affect heart development was described over 50 years ago[2,3], we are only just beginning to appreciate mechanistically how cells interpret the activation of retinoid signaling. Because the mechanistic details of RA biosynthesis and metabolism are becoming more evident, new tools are emerging to dissect the multiple roles for RA signaling further. As the field pushes forward, it has become increasingly clear that some of the effects of retinoid receptors are due to cross-talk with other developmentally relevant signal pathways. Although many of these studies have been performed in noncardiac cell types, it is probable that similar protein interactions exist in the embryo and, as such, are paving the way for the expanding role of retinoid signaling in the developing heart.

## RETINOIC ACID BIOSYNTHESIS AND METABOLISM IN THE DEVELOPING HEART

Dietary vitamin A is biologically inert and must be converted to RAs, which act as ligands for the retinoid X- and RA receptors (RXR and RAR, respectively). Vitamin A is converted to its alcohol derivative retinol, which is then metabolized to retinyl ester, the stored form of RA, by lecithin:retinol acyltransferase (LRAT). Retinol may also be converted to RA by successive oxidative reactions catalyzed by retinol dehydrogenase (RoDH) to form retinal followed by conversion to all-*trans*RA (atRA) by retinal dehydrogenase (RALDH; although RALDH has been widely accepted as the abbreviation for retinal dehydrogenase, the official nomenclature for the RALDH1, RALDH2, and RALDH3 isoforms is aldh1a1, aldh1a2, and aldh1a3, respectively[4,5,6]). It is known that the specific isomers of RoDH and RALDH function in the embryo and are part of the mechanism that controls temporal and spatial retinoid concentrations[7,8,9]. Interestingly, the recently described RoDH-null shows no cardiac phenotype[10]. In contrast, RALDH-deficient embryos possess ventricular chamber defects[11], indicating that RALDH is a critical regulator of retinoid signaling during heart development. Very recent work shows that in a RALDH-independent manner, cytochrome P450 1B1 (CYP1B1), a mono-oxygenase of the cytochrome p450 family, also yields atRA from retinal[12]. CYP1B1 shows a temporal and spatial expression pattern throughout the avian embryo, coincident with known regions of RA signaling, including those that contribute to heart development[12]. However, in contrast to the RALDH homozygous mutant, CYP1B1-null mice show no cardiac phenotype[13], reinforcing the importance of RALDH during development. (The relevant aspects of retinoid signaling and associated cardiac defects in the respective null mice are shown in Fig. 1.)

While several isomers of RA likely exist *in vivo*, the all-*trans* isoform appears to be the major active metabolite in the RA signal pathway[14]. Different isoforms of RA and retinoid receptor ligands show varying half-maximal concentrations ($EC_{50}$), reflective of their binding affinities for the retinoid receptors (Table 1). As 9-*cis*RA has not been physically detected in the embryos of species other than *Xenopus*[15], there is still controversy concerning whether 9-*cis*RA is a true, functional ligand capable of controlling developmental events[16]. In support of the presence of 9-*cis*RA in the embryo, a 9-*cis*RoDH had been described in the mouse embryo[17] and creates a direct route from vitamin A to 9-*cis*RA that does not rely on isomerization of atRA.

It is evident that polyunsaturated, long-chain fatty acids serve as functional ligands for the retinoid receptors as well. These include docosahexaenoic acid (DHA), a fatty acid known to regulate RXR$\alpha$-mediated signaling in the murine central nervous system[18] and human colonocytes[19]. At present, the significance of these fatty acids in developmental processes is unclear; however, their increasing unregulated use as dietary supplements warrants further investigation.

Catabolic breakdown of RA also plays a significant role in regulating the availability of RA by effectively terminating its signaling potential. The cytochrome P-450 enzyme CYP26 is the protein known to oxidize RA into inactive metabolites[20], the most important step in RA catabolism. CYP26 shows a temporal and spatial expression pattern throughout the embryo[9,21], delineating areas where RA signaling is important. Not surprisingly, production of this enzyme is dependent on levels of RA, as its promoter contains an RA response element (RARE)[20]. Embryos null for CYP26 isoforms display a variety of defects, including malformations in cardiac looping and defects associated with abnormal gastrulation and head development[21], whereas maternal supplementation of RA to CYP26 nulls recapitulates the teratogenic potential of excess RA[22].

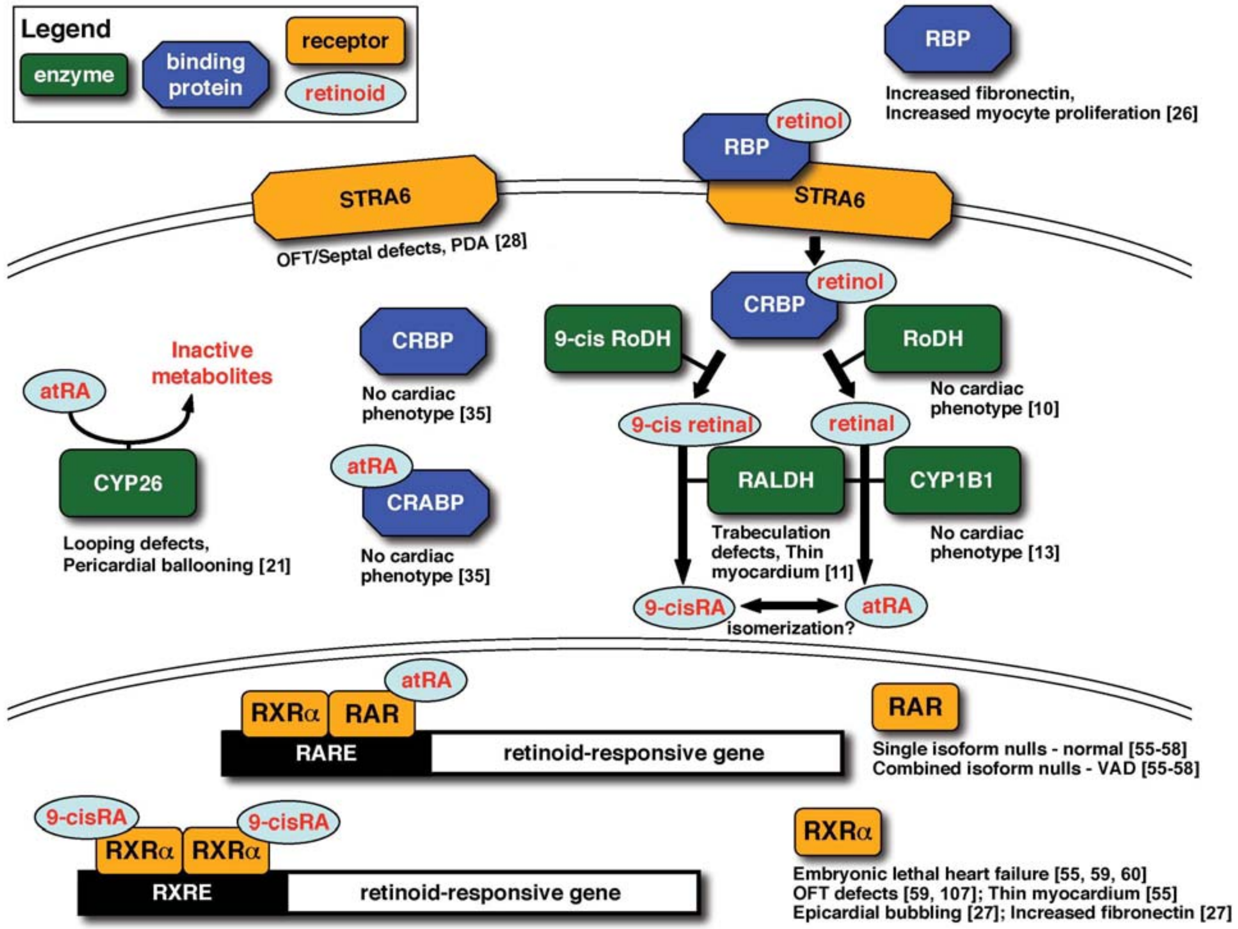

**FIGURE 1.** Retinoid signaling and associated cardiac phenotypes in mutant mice. Green signifies protein is an enzyme; blue, retinoid binding protein; orange, receptor; red font, retinoid. See text for pathway details. Where knockout mice have been generated, a brief description of the phenotype is provided adjacent to the protein along with the appropriate references. No description is given if a knockout has not been reported to date. CYP1B1, cytochrome P450 1B1; CYP26, cytochrome P450 26; RALDH, retinaldehyde dehydrogenase; RoDH, retinol dehydrogenase, 9-*cis*RoDH, 9-*cis* retinol dehydrogenase; RBP, retinol binding protein; CRBP, cellular retinol binding protein; CRABP, cellular retinoic acid binding protein; STRA6, retinol binding protein receptor; RXRα, retinoid X receptor alpha; RAR, retinoic acid receptor; atRA, all-*trans* retinoic acid; 9-*cis*RA, 9-*cis* retinoic acid; RXRE, retinoid X response element; RARE, retinoic acid response element; OFT, outflow tract; PDA, patent ductus arteriosus; VAD, Vitamin A Deficiency Syndrome.

Retinoid binding proteins regulate the availability of retinol and RA in the developing organism. This class of proteins includes the extracellular retinol binding protein (RBP) as well as intracellular retinol binding proteins (CRBPs) and RA binding proteins (CRABPs). All of these proteins play a role in RA signaling by regulating the availability of retinol and RA either within the extracellular spaces or by compartmentalizing available RA inside the cell. RBP is expressed throughout embryogenesis and delivers retinol from external sources to the plasma membrane for uptake via the RBP-specific receptor STRA6[23]. Initial expression of RBP protein occurs before the onset of cardiogenesis. It is then found in the cardiac jelly at the heart tube stage followed by expression in the myocardium slightly later in development[24]. Antisense oligos against RBP decrease RA-dependent activation of a RAR promoter[25] and even though RBP-null mice appear normal when maintained on a diet sufficient in vitamin A, early-age embryos display premature differentiation and increased proliferation of myocytes, as well as increased accumulation of fibronectin[26]. This latter observation may be mediated in part by RXRα as embryos null for this receptor subtype also displayed an elevated fibronectin deposition in several regions of the E9.5–10.5 embryonic heart[27]. Lending clinical significance to the importance of

**TABLE 1**
**Commonly Used Retinoid Receptor Ligands***

| | **RAR** | | | **RXR** | | |
|---|---|---|---|---|---|---|
| | α | β | γ | α | β | γ |
| all-*trans*RA[a,b,c] | 169–436 | 9–78 | 2–19 | 916–1015 | 1211–1492 | 961–1130 |
| 9-*cis*RA[a,b,c] | 13–220 | 29–173 | 45–58 | 195–253 | 128–221 | 124–147 |
| DHA[d,e,f] | | NA | | | 5–100 μM | |
| Phytanic acid[g] | | NR | | NR | 20 μM | NR |
| Bexarotene[h,i,j] | 7–10 μM | 0.29–10 μM | 1.4–10 μM | 25–33 | 24–41 | 20–25 |
| Isotretinoin[c] | 124 | 47 | 36 | | NR | |
| Am80[k,l,m] | 2–46 | 7–235 | 149–591 | | > 5 μM | |

* Half-maximal effective concentration ($EC_{50}$) ranges are reported in nanomolar concentrations unless otherwise noted. Table was compiled based on the indicated references. DHA, docosahexaenoic acid. NA, no activity detected at any concentration. NR, no data reported.

a. Farmer, L.J., Zhi, L., Jeong, S., Lamph, W.W., Osburn, D.L., Croston, G., Flatten, K.S., Heyman, R.A., and Nadzan, A.M. (2003) Retinoic acid receptor ligands based on the 6-cyclopropyl-2,4-hexadienoic acid. *Bioorg. Med. Chem. Lett*. **13(2),** 261–264.

b. Allegretto, E.A., McClurg, M.R., Lazarchik, S.B., Clemm, D.L., Kerner, S.A., Elgort, M.G., Boehm, M.F., White, S.K., Pike, J.W., and Heyman, R.A. (1993) Transactivation properties of retinoic acid and retinoid X receptors in mammalian cells and yeast. Correlation with hormone binding and effects of metabolism. *J. Biol. Chem*. **268(35),** 26625–26633.

c. Idres, N., Marill, J., Flexor, M.A., and Chabot, G.G. (2002) Activation of retinoic acid receptor-dependent transcription by all-trans-retinoic acid metabolites and isomers. *J. Biol. Chem*. **277(35),** 31491–31498.

d. de Urquiza, A.M., Liu, S., Sjoberg, M., Zetterstrom, R.H., Griffiths, W., Sjovall, J., and Perlmann, T. (2000) Docosahexaenoic acid, a ligand for the retinoid X receptor in mouse brain. *Science* **290(5499),** 2140–2144.

e. Goldstein, J.T., Dobrzyn, A., Clagett-Dame, M., Pike, J.W., and DeLuca, H.F. (2003) Isolation and characterization of unsaturated fatty acids as natural ligands for the retinoid-X receptor. *Arch. Biochem. Biophys*. **420(1),** 185–193.

f. Lengqvist, J., Mata De Urquiza, A., Bergman, A.C., Willson, T.M., Sjovall, J., Perlmann, T., and Griffiths, W.J. (2004) Polyunsaturated fatty acids including docosahexaenoic and arachidonic acid bind to the retinoid X receptor alpha ligand-binding domain. *Mol. Cell. Proteomics* **3(7),** 692–703.

g. Zomer, A.W., van Der Burg, B., Jansen, G.A., Wanders, R.J., Poll-The, B.T., and van Der Saag, P.T. (2000) Pristanic acid and phytanic acid: naturally occurring ligands for the nuclear receptor peroxisome proliferator-activated receptor alpha. *J. Lipid Res*. **41(11),** 1801–1807.

h. Hermann, T.W., Yen, W.C., Tooker, P., Fan, B., Roegner, K., Negro-Vilar, A., Lamph, W.W., and Bissonnette, R.P. (2005) The retinoid X receptor agonist bexarotene (Targretin) synergistically enhances the growth inhibitory activity of cytotoxic drugs in non-small cell lung cancer cells. *Lung Cancer* **50(1),** 9–18.

i. Boehm, M.F., Zhang, L., Zhi, L., McClurg, M.R., Berger, E., Wagoner, M., Mais, D.E., Suto, C.M., Davies, J.A., Heyman, R.A., et al. (1995) Design and synthesis of potent retinoid X receptor selective ligands that induce apoptosis in leukemia cells. *J. Med. Chem*. **38(16),** 3146–3155.

j. Howell, S.R., Shirley, M.A., Grese, T.A., Neel, D.A., Wells, K.E., and Ulm, E.H. (2001) Bexarotene metabolism in rat, dog, and human, synthesis of oxidative metabolites, and in vitro activity at retinoid receptors. *Drug Metab. Dispos.* **29(7),** 990–998.

k. Schneider, S.M., Offterdinger, M., Huber, H., and Grunt, T.W. (2000) Activation of retinoic acid receptor alpha is sufficient for full induction of retinoid responses in SK-BR-3 and T47D human breast cancer cells. *Cancer Res.* **60(19),** 5479–5487.

l. Delescluse, C., Cavey, M.T., Martin, B., Bernard, B.A., Reichert, U., Maignan, J., Darmon, M., and Shroot, B. (1991) Selective high affinity retinoic acid receptor alpha or beta-gamma ligands. *Mol. Pharmacol*. **40(4),** 556–562.

m. Nagy, L., Thomazy, V.A., Shipley, G.L., Fesus, L., Lamph, W., Heyman, R.A., Chandraratna, R.A., and Davies, P.J. (1995) Activation of retinoid X receptors induces apoptosis in HL-60 cell lines. *Mol. Cell. Biol*. **15(7),** 3540–3551.

STRA6 is a recent report describing the devastating effects of autosomal-recessive mutations within this gene. Children of consanguineous STRA6 mutation-carrier parents showed ocular, cardiac, and pulmonary defects, as well as profound cognitive deficits[28]. The cardiac defects in individuals carrying point mutations include Tetrology of Fallot, ventricular and atrial septal defects, persistent ductus arteriosus, and aortic arch defects[28].

Intracellular binding proteins for retinol and RA are also expressed throughout development, and their expression patterns within the heart suggest they play important roles during cardiogenesis[29,30, 31,32].CRBP binds RA precursors and presents them to RoDH and RALDH, effectively increasing the cellular pool of RA[20]. CRABP has two isoforms, CRABP1 and CRABP2, with apparently different roles in RA signaling. CRABP2 binds RA as well as nuclear retinoid receptor heterodimers bound to RAREs and increases transcriptional activity[33]. In contrast, CRABP1 is believed to sequester RA in the cytosol and prevent its entry into the nucleus, negatively regulating subsequent transcriptional activity[34]. Interestingly, just as with the RBP-null, mice deficient in the CRBPs or CRABPs appear normal and viable. Even though levels of retinol and retinyl esters are affected by the absence of CRBP, levels of RA are unchanged from normal[35]. Thus, levels of stored retinol and retinyl esters are decreased, but the amount of RA synthesized is not altered nor are target genes for RA affected by the absence of CRBP[35].

The above studies suggest that controlling the biosynthesis of RA and regulating its receptors determine how RA signaling affects cardiac development. Unfortunately, studies examining RA biosynthesis in the developing heart have been limited. In the E10.5–E14.5 whole mouse embryo, levels of retinol, retinyl ester, and RA have been quantitated using HPLC[35]. Although local concentrations of RA may be significant enough to activate RA signaling, the above studies showed that overall levels of RA decrease over these midgestational time periods. To determine whether *in vivo* generation of RA activates receptor-dependent RA signaling, indicator mice have been generated containing RAREs driving β-galactosidase expression. These studies showed that synthesis of RA occurs throughout the E9.5–14.5 embryo, including the heart[36]; however, the predominant area where RA generation takes place is within neural tissues[36,37,38,39]. In separate reports, activation of RA signaling resulted in lacZ expression in the epicardium and subepicardial myocardium[35], as well as the myocardium itself[40]. These results are consistent with reports that suggest that RA signaling is important for epicardium development and for myocardial proliferation and maturation, which presumably originates in the epicardium and epicardially derived cells[27,41,42]. An epicardial role is further supported by recent results that demonstrate an epicardially restricted expression pattern of the RA synthesizing enzyme RALDH2, which indicates regions of endogenous RA signaling[43,44,45,46]. Exogenous administration of RA to the embryo interestingly results in RA signaling in regions where RALDH2 is not found[35,36,39,40]. This may, in part, explain the teratogenic effects of elevated RA, particularly in the outflow tract and ventricular myocardium. Because of the specificity of the tissues responding to endogenously produced RA, these studies also demonstrate that local, rather than circulating, RA is likely the mechanism through which retinoid signaling is activated.

## RETINOIC ACID RECEPTORS

Metabolites of vitamin A mediate their effects through specific RA receptors inside the cell. These receptors are members of a superfamily of nuclear hormone receptors that are ligand-dependent transcription factors[47]. Retinoid receptors are divided into two main families, the retinoic acid receptors (RARs) and the retinoid X receptors (RXRs). Each of these subfamilies contains three subtypes—α, β, and γ, which serve as high-affinity receptors for various vitamin A metabolites. It appears that all the known natural retinoids activate the RARs, while a specific 9-*cis* conformation is required for activation of the RXRs. The biological function of these receptors requires the heterodimerization between the RXRs and other nuclear receptors, such as the RARs, thyroid hormone receptor (TR), and vitamin D receptors. This interaction places RXR at the center of many regulatory processes that either require RA

signaling as part of a normal signal transduction mechanism or simply necessitate the heterodimeric interaction with RXR, which may or may not require ligand[48,49].

Most of the reported expression patterns of retinoid receptors in the heart are from *in situ* hybridization studies. Expression patterns in the early embryo have come from studies examining the precardiac fields and early heart tube in the avian. It appears that all subtypes of retinoid receptors are expressed at this time in the quail embryo[50], although RARα2 is important for inflow tract formation and RARγ is involved in left-right asymmetry and looping[51]. In both instances, it seems that the heterodimer partner is RXRα[51]. In later, midgestational stages in the mouse, RARα, RXRα, and RXRβ transcripts are ubiquitously expressed, although to varying degrees in different lineages, with RXRα being the lowest[52,53]. Transcripts for RARβ, RARγ, and RXRγ display more restricted expression patterns. Of these, the RARγ gene, which is at very low levels in the heart tube, is highly expressed in the endocardial cushions of the midgestation embryo[54]. Interestingly, both RXRα and RXRβ expression are highest in the whole embryo at midgestation (E11.5–12.5), while expression for RXRγ peaks slightly later, around E13.5–14.5 [53]. Protein expression studies in cardiac tissue have been limited.

Even though transcript levels may be nearly undetectable, genetic studies of receptor subtype nulls lend significance to most of the receptor isoforms because of the resultant phenotypes in their absence. Indeed, each receptor subtype has been deleted and knockout mice have been generated. Although embryos that carry specific combinations of the various receptor mutations result in a variety of cardiac defects[55,56,57,58], the only mutation that singly leads to cardiac defects and embryonic lethality is the RXRα subtype[59]. In mice, null mutation for the RXRα gene causes embryonic lethality due to cardiac failure[55,59,60], which may be the result of reduced myocyte proliferation and precocious differentiation[55], as well as loss of NADH ubiquinone reductase activity and impaired oxidative phosphorylation[61]. RXRα has also been removed by cre-flox strategy in several different cell lineages, including ventricular cells expressing MLC2v-cre[62], neural crest cells expressing Pax3-cre[63] and Wnt-1-cre[42], and epicardial-derived cells expressing GATA-5-cre[42]. Of these, the only conditional knockout that demonstrated a phenotype was the GATA-5-cre transgene, which is expressed predominantly in epicardial cell lineages, including the body wall, as well as limited expression in the adjacent myocardium[42]. In these mice, the ventricular phenotype closely resembled the systemic RXRα null[59], and since the MLC2v-cre/RXRα-flox mouse displayed no phenotype, it is reasonable to conclude that normal communication between myocardium and epicardium during cardiac development relies on appropriate RA signaling within the developing epicardium.

## RETINOIC ACID SIGNALING IN THE PRECARDIAC FIELDS AND EARLY HEART TUBE

The precardiac fields, which lie in the lateral plate mesoderm of the early embryo on either side of Henson's node, are profoundly influenced by factors that establish left-right asymmetry. Local sources of RA in this region may influence how the subsequent heart tube develops and is exemplified by the differential sensitivity of the left and right precardiac fields to RA exposure[64]. In the chick embryo, RA exposure in regions of precardiac mesoderm that normally are not exposed to RA can randomize looping and cause situs inversus and cardia bifida[65]. A similar spectrum of malformations is observed in chick and quail embryos under vitamin A deficiency[1,66,67]. In fact, vitamin A deficiency is lethal to avian embryos[68,69], and results in absence of both RA and retinoid receptors in the heart-forming region[70] and in a failure to link extraembryonic vasculature with the inflow tract[1,66]. The anterior-posterior axis may also be modulated by retinoid signaling in that RA treatment causes expansion of atrial-specific gene expression into ventricular-fated cells[71]. *Xenopus* embryos exposed to a RAR antagonist show dose-dependent effects on early cardiac development. At low doses, a heart tube forms, but shows looping defects. Intermediate and high doses of antagonist yield loss of tube formation and lack of lateral myocardial fusion, respectively[72]. In zebrafish embryos, RA is restricted to the cardiac progenitor pool, effectively decreasing the number of embryonic multipotential cells that will contribute to heart development without affecting subsequent proliferation[73]. Conversely, treatment with a pan-RAR antagonist expanded the cardiac progenitor pool as assessed by Nkx2.5 and MLC2 expression[73]. Downstream effectors in the RA signal pathway that control these events are not well understood, although GATA-4 may be involved since it is down-regulated in the vitamin A–deficient quail model[74] and up-regulated in the presence of excess retinoids[75]. It was further suggested that excess GATA-4 antagonizes transcription of Nkx2.5 in cardiac precursor regions, ultimately leading to a lack of cardiomyogenesis[75].

The anterior-secondary heart-forming field (AHF), identified simultaneously by three groups[76,77,78], has become the focus of a rapidly evolving field of research. This region of pharyngeal mesoderm contributes myocardium to the OFT, and ablation of this field in the chicken at Hamburger-Hamilton stage (HH) 14 results in variably penetrant overriding aorta and pulmonary atresia[79]. Ablation at HH18 yields coronary artery defects[79]. The importance of retinoid signaling in the AHF remains to be determined as direct investigations of interactions have not been reported and many of the landmark retinoid teratogenicity studies were performed before the AHF was identified. It has been hypothesized, however, that since AHF cells are in close proximity to the cardiac crescent at late gastrulation, they are exposed to similar RA signals[80]. Arguing in favor of the importance of retinoid signaling in the AHF are models of attenuated and excessive retinoid signaling, which often show similar phenotypes to those seen when the AHF is perturbed. Indirect evidence suggests that interactions exist between retinoid signaling in the AHF at the level of Islet1, a purported AHF-specific marker. For example, agonized RXR$\alpha$ promotes degradation of $\beta$-catenin[81], a positive regulator of Islet1[82]. Alternately, loss of Islet1 leads to the down-regulation of FGF and BMP[83], which are known retinoid target genes. These studies suggest that a signaling loop exists between retinoids and Islet1, and will undoubtedly pave the way for understanding the role of retinoid signaling in the contribution of the AHF to the developing heart. Collectively, these studies demonstrate that proper retinoid signaling is indispensable for normal heart development even prior to heart tube formation.

## RETINOIC ACID SIGNALING IN THE DEVELOPMENT OF THE EPICARDIUM AND ASSOCIATED EFFECTS ON VENTRICULAR MATURATION

It is clear that intact retinoid signaling is essential for proper epicardial development and, secondarily, for the downstream morphological events that are dependent on epicardially derived cells. In the murine embryo around E9.5, epicardially derived cells from the proepicardium, a cluster of extracardiac mesothelial cells residing in the coelomic cavity just caudal to the atria, migrate and proliferate over the surface of the heart forming the epicardial layer. The epicardium and epicardially derived cells contribute in multiple ways to heart development, including the formation of the coronary arteries, portions of the conduction system, and interstitial fibroblasts (for recent reviews see [84,85,86]). During each stage of cardiac development involving the epicardium, retinoid signaling has been implicated. In both avians and mice, RALDH2 is expressed in the proepicardium and continues to be expressed in the migratory epicardial cells[43,44,45,46] as well as in endocardial cells overlying the atrioventricular cushions and in the developing mouse pericardium (Fig. 2). These expression patterns reveal the populations of cells that are responsible for local retinoid synthesis. It is believed that RA produced by the migrating epicardial cells acts on the ventricular myocardium in a paracrine manner[43] and that this retinoid signal is necessary for proper ventricular maturation.

In the chick model, epicardially derived cells expressing RALDH2 are contained within the subepicardial space before invading the ventricular myocardium[45]. Once within the ventricular myocardium, these cells differentiate into smooth muscle, endothelial, and interstitial fibroblast cells at which time their expression of RALDH2 is down-regulated[45]. RALDH2 knockout mice have heart defects including improper ventricular trabeculation secondary to decreased cardiomyocyte proliferation, underscoring the importance of epicardial RA production[11]. Ventricular maturation takes place during

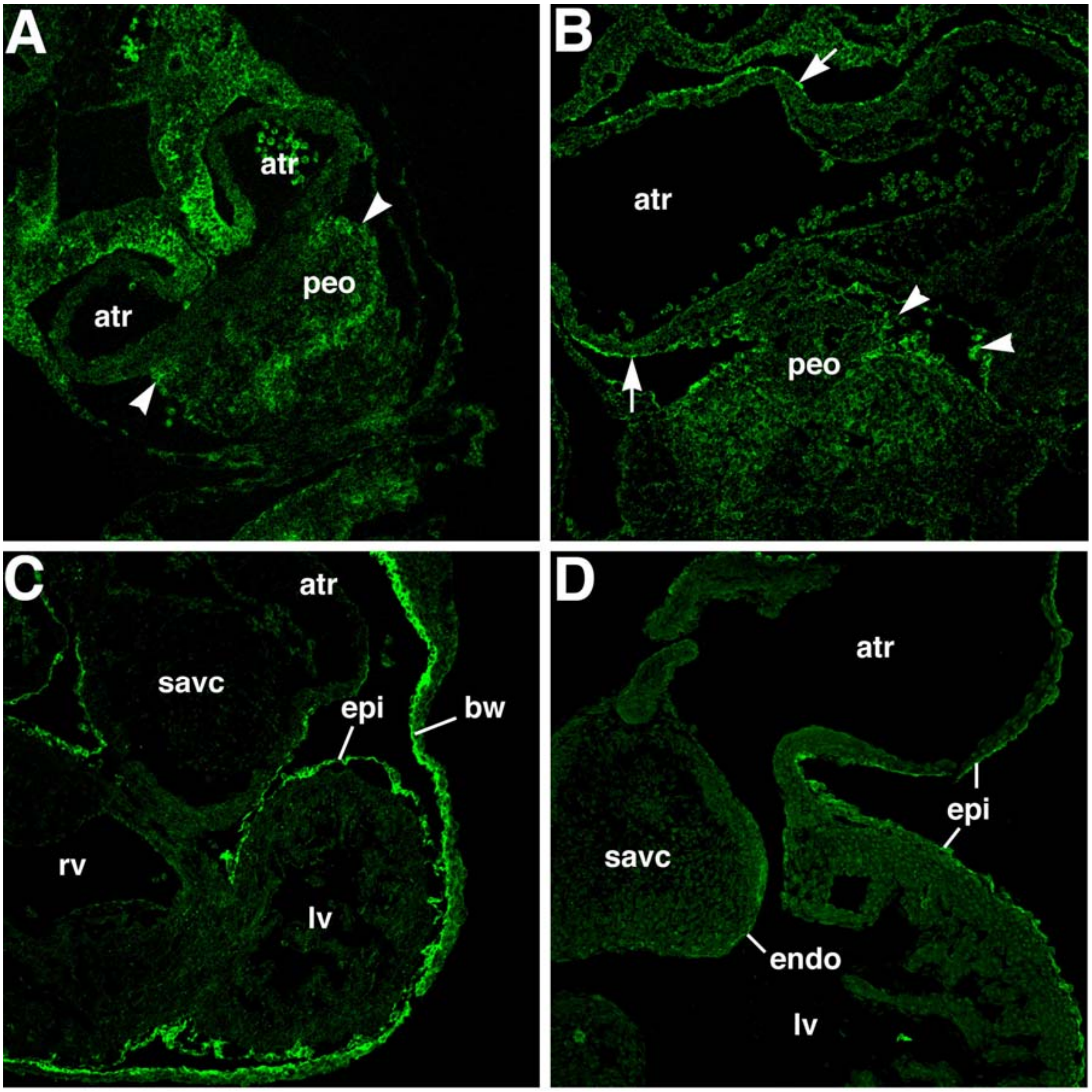

**FIGURE 2.** RALDH2 immunolocalization in the embryonic mouse heart labels both proepicardium and epicardium. Sections from embryonic day 9.5 (A), 10.5 (B), 11.5 (C), and 12.5 (D) mouse embryos immunostained against RALDH2 show immunolocalization in the proepicardium (peo) at E9.5 and E10.5 (panel A and B, arrowheads), as well as newly formed epicardium at E10.5 (panel B, arrow). Note that regions of myocardium devoid of proepicardium-derived cells do not express RALDH2. By E11.5, the heart is covered with epicardium (epi) and expresses RALDH2 over its entire surface. RALDH2 immunolocalization is also observed in an epithelial layer adjacent to the body wall (bw) and likely represents the developing pericardium. RALDH2 continues to be expressed in the epicardium and subepicardial cells at E12.5 (panel D), as well as in the endocardium (endo) over the atrioventricular valve primordia.

midgestation, so it is important to note that the expression of an RARE-lacZ transgene in the epicardium and in the subepicardial myocardium suggests that RA signaling also takes place in this region of the heart at E12.5[36].

There is increasing evidence to show that one particular retinoid receptor isoform, RXRα, plays a pivotal role in epicardial RA signaling and in ventricular maturation. The RXR null ($RXR\alpha^{-/-}$) embryo shows a delay in the proepicardium-epicardium transition with several areas of bare myocardium existing up until E11.5[27]. The epicardium that is formed in the mutant has abnormally large subepicardial spaces between the epicardium and myocardium, resulting in a defect[27] termed epicardial “bubbling”.

There is also elevated apoptosis, and disorganized and elevated fibronectin within the proepicardium, both of which may contribute to the aberrant cardiac phenotype of the $RXR\alpha^{-/-}$[27]. Additionally, the expression of vascular cell adhesion molecule-1 (VCAM1) mRNA[87] and protein (Burton, Hoover, and Kubalak, unpublished observations) are reduced in the $RXR\alpha^{-/-}$. VCAM1 is a membrane-bound protein important for cell-cell adhesion and cell-extracellular matrix association. Consequently, a reduction in VCAM1 would explain the epicardial phenotype in the $RXR\alpha^{-/-}$. The VCAM1 knockout is embryonic lethal, lacks an epicardium, and shows a thin myocardial wall indicating a role for VCAM1 in both epicardial and myocardial development[88,89]. The link between signaling through RXRα and expression of VCAM1 is currently under investigation.

Several aspects of the $RXR\alpha^{-/-}$ mouse heart phenocopy the vitamin A–deficient rat. For example, both models lack a properly formed cardiac compact zone secondary to reduction in the proliferative rate of ventricular cardiomyocytes, resulting in a thin hypoplastic ventricle[3,87,90,91]. The current hypothesis is that the epicardium secretes a mitogenic factor that promotes compact zone expansion and this process is dependent on intact retinoid signaling within the epicardium[41,92].

In order to determine the cardiac cell lineage most vulnerable to loss of RXRα, conditional knockouts of RXRα have been generated within several cell lineages. Ventricular myocyte loss of RXRα did not result in significant cardiac abnormalities[62], whereas epicardial loss of RXRα showed a similar cardiac phenotype as that seen in the germline knockout[42]. Specifically, loss of RXRα in the epicardium results in a thinning of the myocardial wall and, although not reported in the systemic knockout, displays defects in coronary arteriogenesis[42]. In these conditional knockouts, there is a down-regulation of several genes implicated in retinoid signaling, namely FGF2, β-catenin, and Wnt9b[42]. *In vitro* work showed FGF is mitogenic for cultured cardiomyoblasts[93,94,95], and more recently, it was discovered that epicardially derived FGF signals aid in myocardial proliferation[96] and serve as a modulator for epicardial epithelial-mesenchymal transformation[97,98]. Activation of the Wnt/β-catenin pathway regulates several processes during heart development, including formation and proliferation of cardiomyocytes[99,100], epithelial-mesenchymal transformation of endocardial cells[101], and regulation of vasculogenesis[102]. (For comprehensive reviews of Wnt signaling during cardiac development see [103,104]). Overall, these studies suggest that controlled retinoid signaling within the epicardium is critical for proper cardiac remodeling, although much work must be done to determine the exact molecular mechanisms involved.

## RETINOIC ACID SIGNALING IN ENDOCARDIAL CUSHION DEVELOPMENT

Proper formation and remodeling of the endocardial cushions is required for normal valve development, as well as chamber and outflow tract septation, and it is clear that retinoid signaling plays an important role in regulating these processes. Embryonic day 10 embryos exposed to excess RA via maternal gavage at E9 showed undersized atrioventricular cushions due to decreased extracellular matrix and fewer populating mesenchymal cells[105]. Extracellular matrix constituents and their patterns of expression in embryos exposed to excess RA showed aberrant fibronectin and collagen-1 expression[106] and blunted endocardial epithelial-mesenchymal transformation in the outflow tract[56]. Because cardiac neural crest cells migrate into the endocardial cushions, and excess RA can perturb their migration (see below), the exact etiology of the loss of endocardial cushion mesenchymal cells is still unclear.

These results are in contrast to the initial development of cushion tissue in the $RXR\alpha^{-/-}$ embryo where cushion sizes and mesenchymal cell numbers are normal at E11.5. However, by E12.5, they display hypoplastic endocardial cushions as a result of increased cushion cell apoptosis[107]. This mutant embryo shows elevated levels of the proapoptotic molecule transforming growth factor β2 (TGFβ2), which is implicated in the increased cushion cell death[107]. It appears, therefore, that RA can regulate endocardial epithelial-mesenchymal transformation in an RXRα-independent manner while expansion and maturation of cushion tissue requires signaling through RXRα.

## RETINOIC ACID SIGNALING IN NEURAL CREST CELLS AND AORTICOPULMONARY SEPTATION

Cardiac neural crest cells (CNCC), a subpopulation of cranial neural crest cells, migrate into and populate specific areas of the embryonic heart. Aberrant migration and disruption of signaling pathways in these cells can lead to defects in outflow tract septation and result in persistent truncus arteriosus, as well as other related aortic arch defects. It was first noted that ablation of CNCC can cause phenotypes similar to those seen in embryos exposed to high amounts of retinoids, a condition known as RA embryopathy[108]. Further studies have shown that the teratogenic effects of RA are dose dependent and affect all structures that are populated by the neural crest cells[109]. Embryos of pregnant dams administered RA via gavage showed a 40% decrease in neural crest cell migration and a decrease in cell proliferation[110]. Similarly, neural crest cells treated with RA *in vitro* showed a dose-dependent attenuation of cell migration and decreased cell proliferation[110]. Further characterization showed that the effect of RA on neural crest cell behavior might be secondary to inhibition of PDGF-mediated cell proliferation and migration via blockade of JNK activation[110].

Migrating neural crest cells do not express RALDH2[7], the primary enzyme that converts retinal to RA. Conversely, RALDH2 is expressed throughout the embryonic heart during the same developmental time frame[7] leading to the theory that the effects of RA on CNCC occur in a paracrine manner. The importance of early RA signaling on CNCC is suggested by a study in E6.25–E10.25 embryos showing that CNCC migration is aberrant in RALDH2 knockouts that are otherwise rescued by maternal RA administration[11]. RALDH2 knockout embryos die *in utero* at E10.5 due to heart failure as a result of cardiac looping defects, lack of ventricular trabeculation, and poor outflow tract septation. Upon maternal administration of RA prior to E10.5, many of the cardiac defects are reversed, allowing for survival until midgestation; however, conotruncal defects remained. Further analysis showed that even upon rescue with RA, CNCC did not properly migrate into and populate the outflow tract[11]. At least part of this phenotype may be due to influences of RA within the migratory pathway of neural crest cells by the surrounding pharyngeal arch tissues[111]. Lack of RA signaling as in the RALDH2 hypomorph results in a down-regulation of RA-responsive genes and underdeveloped pharyngeal arches 3 through 6, which may have a profound influence on neural crest cell migration[111].

The importance of retinoid signaling in the CNCC has been further investigated in models of vitamin A deficiency and in retinoid receptor knockout mice[112,113,114]. RAR/RXR double mutants and RXRα knockouts show conotruncal defects similar to those seen in vitamin A deficiency models and it is believed that these observations are either the result of aberrant CNCC migration[112] or altered signaling in resident CNCCs[113]. Defects in vitamin A–deficient embryos are rescued by exogenous RA when administered at a time coincident with CNCC arrival in the heart (E9.5–10.5)[2]. However, there remains controversy regarding the role for retinoid signaling in neural crest cells since others have shown that RA failed to rescue the CNCC-related phenotype in the RALDH2 null[11]. Thus, it is likely that more thorough studies of RA rescue in these models will unravel the mechanistic role for retinoid signaling in the CNCC.

## THE FUTURE: INTERACTIONS BETWEEN RETINOID SIGNALING AND OTHER DEVELOPMENTALLY RELEVANT SIGNAL PATHWAYS

Future research avenues regarding retinoid signaling during cardiovascular development most certainly will include the study of interactions between retinoid signaling and other molecular pathways. It is clear that activation of retinoid signaling impacts other pathways by regulating the transcription of a variety of genes. Retinoids can serve as molecular sensors for the up- or down-regulation of other developmentally significant pathway mediators, such as Homeobox transcription factors[115], Wnt signaling axis members[116], and as described below, TGFβ. Additionally, the transcriptional repression of certain gene products by retinoid receptors in the absence of ligand (i.e., without RA isoforms) serves a role that is as important as positive transcriptional regulation by liganded receptors. This is exemplified by direct interactions between retinoid receptors and the signaling cofactors directly downstream of TGFβ, the Smads. Thus, while the classical mechanism of regulation by retinoids via formation of heterodimeric transcriptional complexes is well described, new evidence suggestive of an unliganded role for retinoid receptors is emerging.

It is well established in various model systems that retinoid and TGFβ signal pathways reciprocally regulate each other at the transcriptional level. For example, treating keratinocytes and leukemia cells with atRA, an RAR agonist, increases the production of TGFβ2[117], while other TGFβ isoform levels remain unchanged[118]. In the embryonic system, however, the general consensus is that exogenous retinoids lead to the down-regulation of TGFβ1 and TGFβ2[119]. However, there are reports of exogenous RA exposure resulting in both up- and down-regulation of TGFβ2 mRNA and protein depending on the cell type[120].

Several lines of evidence suggest that interactions between RA and TGFβ signaling play important roles during cardiac development. Administration of RA to pregnant dams at E8 resulted in ubiquitous down-regulation of TGFβ2 throughout the mouse embryo. Intracellular TGFβ1 was reduced in all tissues except the myocardium, while extracellular TGFβ1 was reduced in the CNCC and neuroepithelium[119]. In contrast, embryos homozygous null for RALDH2 show a down-regulation of TGFβ1 that can be rescued with exogenous RA treatment[121]. Not surprisingly, reciprocal effects of TGFβ signaling on retinoids also occur. For example, in MC3T3-E1 osteoblast cells, TGFβ1 is known to transcriptionally up-regulate levels of RARα RARγ, and RXRα through TGFβ1-mediated up-regulation of the transcription factors c-fos and c-jun[122]. This results in a TGFβ-mediated increase in retinoid signaling via RAR-RXR heterodimers. Whether these latter regulatory responses occur in the heart during development remains to be established.

Loss of normal retinoid signaling as in the $RXR\alpha^{-/-}$ embryo results in profound effects on cardiac development, and some of these effects are possibly due to altered TGFβ signaling. TGFβ2 is up-regulated in the midgestational heart in the absence of RXRα[107], suggesting that intact retinoid signaling normally serves to limit the transcription of TGFβ2. A recent study in lung bud development supports this hypothesis in that several TGFβ genes were up-regulated and Smad2 phosphorylation was elevated in RA-deficient foregut tissue[123]. In the $RXR\alpha^{-/-}$, the elevated levels of TGFβ2 mediated, at least in part, the increased apoptosis noted in the outflow tract of the E11.5–13.5 heart. This finding was supported by results in $RXR\alpha^{-/-}$ embryos that were also heterozygous for TGFβ2 ($TGF\beta2^{+/-}$). The resultant phenotype in $RXR\alpha^{-/-}/TGF\beta2^{+/-}$ embryos was a partial rescue of both the apoptosis and the morphologic abnormalities seen in the $RXR\alpha^{-/-}$[107]. These studies demonstrate that a significant degree of cross-talk exists between retinoid and TGFβ signaling during cardiac development.

Recent evidence suggests that at least part of this cross-talk occurs at the level of the immediate downstream signaling molecules in the TGFβ signaling cascade, the Smad family of cofactors, rather than at the level of transcription (see below and Hoover, Burton, and Kubalak, unpublished observations). One report demonstrates that atRA mediates the activity of a Smad2 phosphatase leading to lower levels of activated (phosphorylated) Smad2[124]. Further work, however, is needed to determine the interactions between a recently described Smad phosphatase PPM1A[125] and retinoid signaling. Unliganded RAR can bind to Smad3 and increase Smad3-driven transcription[49]; however, it is unknown if such interactions are at work during embryonic development. Additionally, the transcriptional corepressor TGIF regulates Smad2- and RXR-driven transcription[126]. During high levels of TGFβ signaling indicated by increased nuclear phosphorylated Smad2, TGIF binds Smad2 and prevents its interaction with the transcriptional coactivators CBP and p300, effectively blunting Smad-mediated transcription[127,128]. Under conditions of decreased nuclear phosphorylated Smad2, TGIF binds RXR response elements and blocks the transcriptional activity of RXR homodimers[129]. These interactions may be embryologically significant because loss of function mutations in TGIF have been linked to holoprosencephaly in humans[130,131].

Retinoid signaling has been implicated in other signal pathways as well. For example, RXRα directly interacts with both GATA-4 and FOG-2 to repress transcription in rat neonatal ventricular myocytes[132]. The interaction between RXRα and GATA-4 was independent of the presence of ligand 9-*cis*RA, but the interaction with FOG-2 was dependent on 9-*cis*RA[132]. Additionally, aberrant retinoid signaling may be partially responsible for the teratogenic effects of HMG-CoA reductase inhibitors (i.e., statins) on the embryo, particularly in the cardiovascular system[133]. Cholesterol byproducts known as oxysterols were found to act as ligands for transacting factors controlling the transcription of RALDH1 and RALDH2[133]. This study did not directly show that levels of retinoids were altered in statin-treated systems; therefore, more work is needed to determine if retinoid signaling is indeed reduced or if other retinoid generating mechanisms, such as CYP1B1, are simultaneously up-regulated by these cholesterol-lowering agents.

Other studies investigating how the genetic background of an individual plays a role in retinoid sensitivity are ongoing. For example, heterozygosity for TGFβ2 results in a partial rescue of the cardiac defects seen in the $RXR\alpha^{-/-}$ mouse[107], while total loss of TGFβ2 results in increased sensitivity to the teratogenic effect of exogenous retinoids[134]. Such work may explain the variable teratogenic responses seen in humans exposed to exogenous retinoids[135].

It is clear that excess exogenous retinoids are teratogenic and their availability must be regulated. This regulation is increasingly complicated by the recent development of novel retinoid-based therapeutics for common diseases, such as acne vulgarus, psoriasis, and malignant neoplasm[136,137,138]. Unfortunately, these drugs have and will continue to be misused, leading to increased risk of congenital heart defects. Thus, it is critically important to understand the molecular mechanisms leading to the detrimental effects that these retinoids have on the developing organism. Additionally, these investigations must include the potential affects retinoid signaling may have with other signal pathways. The lessons learned from these studies will undoubtedly bring us closer to the advent of medical interventions that can rescue a developmentally perturbed heart.

## ACKNOWLEDGMENTS

This work was supported by NIH Grant Number C06 RR018823 and C06 RR015455 from the Extramural Research Facilities Program of the National Center for Research Resources, NIH T32 HL07260 (LLH), and NIH/NHLBI R01-HL83116 (SWK).
